# Long-term television viewing patterns and gray matter brain volume in midlife

**DOI:** 10.1007/s11682-021-00534-4

**Published:** 2021-09-06

**Authors:** Ryan J. Dougherty, Tina Hoang, Lenore J. Launer, David R. Jacobs, Stephen Sidney, Kristine Yaffe

**Affiliations:** 1 Department of Epidemiology, The Johns Hopkins Bloomberg School of Public Health, 2024 E. Monument St., Suite 2-700, Baltimore, MD 21205, USA; 2 San Francisco VA Health Care System, San Francisco, CA, USA; 3 National Institute on Aging, Bethesda, MD, USA; 4 University of Minnesota, Minneapolis MN, USA; 5 Kaiser Permanente Division of Research, Oakland, CA, USA; 6 University of California, San Francisco, San Francisco, CA, USA

**Keywords:** Lifestyle factors, Sitting time, Volumetric MRI, Epidemiology, Cohort studies

## Abstract

The purpose of this study was to investigate whether long-term television viewing patterns, a common sedentary behavior, in early to mid-adulthood is associated with gray matter brain volume in midlife and if this is independent of physical activity. We evaluated 599 participants (51% female, 44% black, mean age 30.3 ± 3.5 at baseline and 50.2 ± 3.5 years at follow-up and MRI) from the prospective Coronary Artery Risk Development in Young Adults (CARDIA) study. We assessed television patterns with repeated interviewer-administered questionnaire spanning 20 years. Structural MRI (3T) measures of frontal cortex, entorhinal cortex, hippocampal, and total gray matter volumes were assessed at midlife. Over the 20 years, participants reported viewing an average of 2.5 ± 1.7 hours of television per day (range: 0-10 hours). After multivariable adjustment, greater television viewing was negatively associated with gray matter volume in the frontal (β = −0.773;*p* = 0.01) and entorhinal cortex (β = −23.8;*p* = 0.05) as well as total gray matter (β = −2.089; *p* = 0.003) but not hippocampus. These results remained unchanged after additional adjustment for physical activity. For each one standard deviation increase in television viewing, the difference in gray matter volume z-score was approximately 0.06 less for each of the three regions (*p* < 0.05). Among middle-aged adults, greater television viewing in early to mid-adulthood was associated with lower gray matter volume. Sedentariness or other facets of television viewing may be important for brain aging even in middle age.

## Introduction

While it is generally accepted that a physically active lifestyle is important for cognitive and brain health (Barnes & Yaffe 2011; Dougherty et al. 2016; Dougherty et al. 2021; Dougherty et al. 2017; Erickson et al. 2014; Hamer & Chida 2009; Yaffe et al. 2001), sedentary behavior has increasingly become a public health focus due to evidence that it may impart unique risk for chronic diseases (Dunstan et al. 2012; Katzmarzyk et al. 2009). Sedentary behaviors are defined as any waking behavior characterized by low levels of energy expenditure, while in a seated, reclined or lying posture (Ainsworth et al. 2000). Although a few studies have investigated sedentary lifestyle on cognitive function and dementia risk in older adults (Barnes & Yaffe 2011; Falck et al. 2017; Wheeler et al. 2017), much less is known how sedentary behaviors influence measures of brain health in midlife (Voss et al. 2014).

Reduction in gray matter volume measured by brain magnetic resonance imaging (MRI) is a marker of brain health that often precedes cognitive impairment (Iturria-Medina et al. 2016; Jack et al. 2018). Gray matter declines (i.e. atrophies) during midlife (Raz et al. 2005; Resnick et al. 2003) and longitudinal patterns of atrophy predict future cognitive function and dementia onset (Allison et al. 2019; Driscoll et al. 2009). Therefore, strategies that preserve gray matter volume in midlife may mitigate the progression of cognitive decline. The few recent studies on the effects of sedentary behavior on structural brain outcomes have reported mixed findings, which may be due to differences in definition of sedentary behavior and lack of adjustment for physical activity (Arnardottir et al. 2016; Raichlen et al. 2019; Siddarth et al. 2018). Further, most studies have been cross-sectional, and no study has investigated long-term patterns of sedentary behavior. Therefore, the purpose of this study was to examine the association between television viewing patterns over 20 years with gray matter volume in midlife. We hypothesized that greater long-term television viewing sedentary behavior would be associated with lower gray matter volume at midlife, independent from physical activity.

## Methods

### Participants

The Coronary Artery Risk Development in Young Adults (CARDIA) is a longitudinal study which began in 1985-1986 (year 0) when 5,115 participants (18-30 years old) were recruited by equal distribution of sex, age, education, and race in four U.S. cities (Birmingham, AL; Chicago, IL; Minneapolis, MN; and Oakland, CA). Participants have completed several follow-up visits over 25 years: 1987-1988 (year 2), 1990-1991 (year 5), 1992-1993 (year 7), 1995-1996 (year 10), 2000-2001 (year 15) 2005-2006 (year 20) and 2010-2011 (year 25). During the year 25 exam, a subset of participants underwent magnetic resonance imaging (MRI) as part of the CARDIA Brain MRI ancillary study. The sample for the MRI study were balanced within four strata of ethnicity/race (black, white) and sex from three of the CARDIA field centers (Birmingham, AL; Minneapolis, MN; and Oakland, CA). Additional study recruitment and design details have been previously described (Friedman et al. 1988; Hughes et al. 1987). The CARDIA study and brain MRI ancillary study were approved by the Institutional Review Board (IRB) of each of the participating sites as well as the University of Pennsylvania and University of California, San Francisco. At each examination participants were provided and signed a separate written informed consent for the CARDIA study and the brain MRI ancillary study.

### Television viewing

An interviewer-administered questionnaire was used to determine the number of hours viewing television at years 5, 10, 15, 20, and 25 visits. Participants were asked the average number of hours per day spent viewing television over the previous 12 months. Because television viewing patterns have shown to be stable over time (Yang et al. 2019), we calculated the mean television viewing time over the 20-year period for each participant to assess long-term patterns of television viewing time as a proxy for sedentary activity (Hoang et al. 2016).

### Neuroimaging protocol

At year 25, MRI scans were acquired in the axial plane on 3T scanners located at each CARDIA site; a Siemens 3T Tim Trio/VB 15 platform in Minneapolis and in Oakland; a Philips 3T Achieva/2.6.3.6 platform in Birmingham. Standard quality assurance protocols which were previously developed for the Functional Bioinformatics Research Network (FBIRN), and the Alzheimer’s disease Neuroimaging Initiative (ADNI) were used with the following thresholds: FBIRN—Siemens scanners Signal-to-Fluctuation-Noise-Ratio (SFNR) > 220, Radius of Decorrelation (RDC) > 3.1, Philips scanners SFNR > 220, RDC > 2.4; ADNI— Signal-to-Noise-Ratio (SNR) > 300, Maximum Distortion > 2.0. The structural images were acquired with 3D T1 and T2 sequences. Scan acquisition parameters have been previously described (Launer et al. 2015), and were processed using previously described methods (Goldszal et al. 1998; Shen & Davatzikos 2002; Zacharaki et al. 2008). In brief, structural images were processed using an automated multispectral computer algorithm which classified all supratentorial brain tissue into gray matter, white matter, and cerebral spinal fluid. After correction of intensity inhomogeneities (Tustison et al. 2010) a multi-atlas skull stripping algorithm was applied for the removal of extra-cranial tissues (Doshi et al. 2013). Each T1-weighted scan was automatically segmented into a set of anatomical gray matter regions of interest (ROIs) using a mutli-atlas label fusion method, MUSE (Doshi et al. 2016). For our analyses, we chose *a priori* gray matter ROIs that have shown to be vulnerable to age-related atrophy including frontal cortex, entorhinal cortex, hippocampal, and total gray matter volumes. Intracranial volume was the sum of gray matter, white matter, and cerebral spinal fluid volumes.

### Covariate measures

All participants completed a variety of health-related questionnaires and measurements at the year 25 visit. Variables that were investigated as potential confounding variables include age, sex, race, years of education, intracranial volume, study site, smoking status, alcohol consumption, body mass index, blood pressure, depression, and physical activity. *Smoking status* and *alcohol consumption* were determined by self-report. *Body mass index* was calculated from the participants measured height and weight (kg/m^2^). *Depressive symptoms* were measured with the Center for Epidemiologic Studies Depression scale (CES-D) (L.S. Radloff 1977). *Physical activity* was evaluated by the CARDIA physical activity questionnaire which assessed vigorous intensity (e.g. jogging & racket sports), moderate intensity (e.g. non-strenuous sport, hiking, home exercising & gardening), and work-related (e.g. heavy lifting on the job) activities over the previous year (Sidney et al. 1991). Scores were converted into total physical activity intensity scores which reflect the estimated number of kilocalories expended per activity, and expressed in exercise units (Jacobs et al. 1989).

### Statistical analyses

Baseline characteristics were compared by television viewing with ANOVA tests, Kruskal-Wallis tests, or χ2 tests, as applicable. Pearson correlations were used to determine the association between television viewing and physical activity. Multivariable linear mixed models were used to test the association between television viewing and gray matter volume. A series of unadjusted and adjusted models were performed to control for the effects of demographics, lifestyle behaviors, and medical co-morbidities. Model 1: adjusted for age, sex, race, education, intracranial volume, and site. Model 2: additional adjustment for physical activity. Sensitivity analysis were conducted with adjustment for smoking status, alcohol consumption, body mass index, blood pressure, depression in addition to those in Model 2. Further, the interactions of race, sex, and age were tested. All statistical analyses were conducted using SAS, version 9.4.

## Results

**Figure 1:**
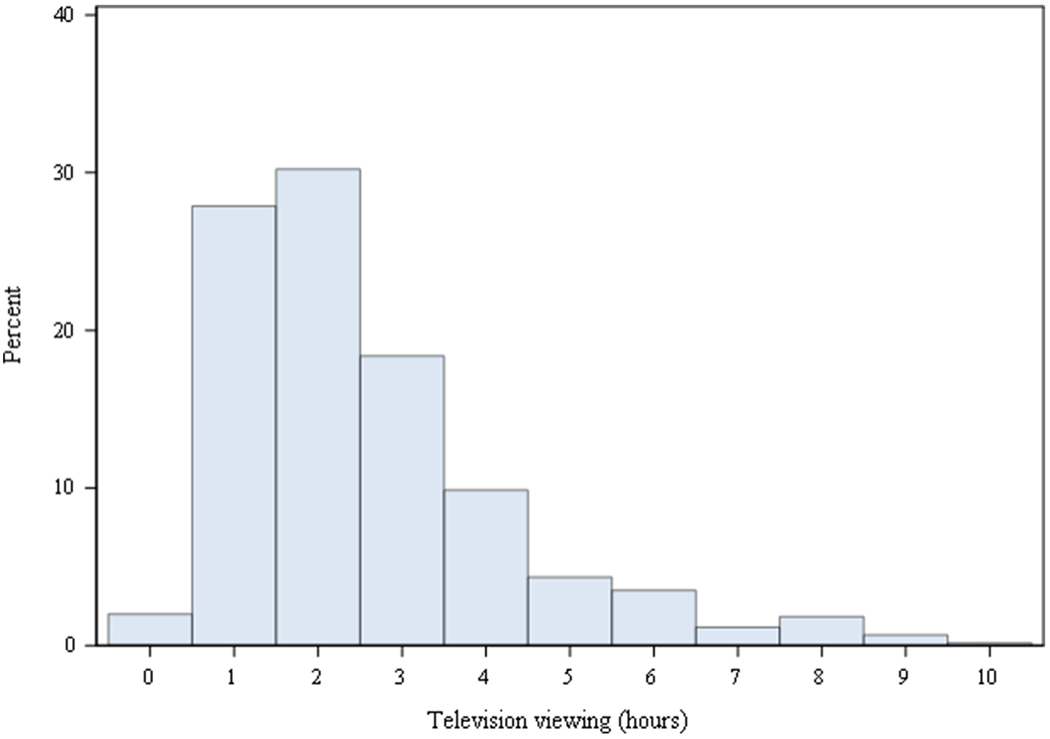
Distribution of television viewing. Average daily television viewing time over the 20-year period

A total of 599 participants (51% female, 44% black, mean age 30.3 ± 3.5 at baseline and 50.2 ± 3.5 years at follow-up and MRI) with longitudinal television viewing and MRI were included in the study. The average number of television viewing assessments was 4.8 ± 0.5 collected over the 20-year period. On average, participants engaged in 2.5 ± 1.7 hours/day of television viewing (range: 0 – 10; Figure 1), and television viewing time was negatively associated with physical activity level (*r* = −0.188; *p* < 0.001). Participants had an average brain volume of 182.127 ± 21.235 ml for frontal cortex, 4494.85 ± 612.907 mm^3^ for entorhinal cortex, 7594.74 ± 840.608 mm^3^ for hippocampal, 634.824 ± 66.117 ml for total gray matter, and 1359.008 ± 153.684 ml for intracranial volume. Additional participant characteristic data are detailed in Table 1.

In the unadjusted models, greater television viewing was negatively associated with all gray matter regions investigated including the frontal cortex, entorhinal cortex, hippocampal, and total gray matter volumes (all *p* < 0.01; Table 2). After adjusting for age, sex, race, education, intracranial volume, and study site (Model 1), the association between television viewing and gray matter volume remained significant for frontal cortex (β = −0.773; *p* = 0.01), entorhinal cortex (β = −23.8; *p* = 0.05), and total gray matter (β = −2.089;*p* = 0.003). These results remained unchanged after additional adjustment for total physical activity (Model 2; Table 2).

**Figure 2:**
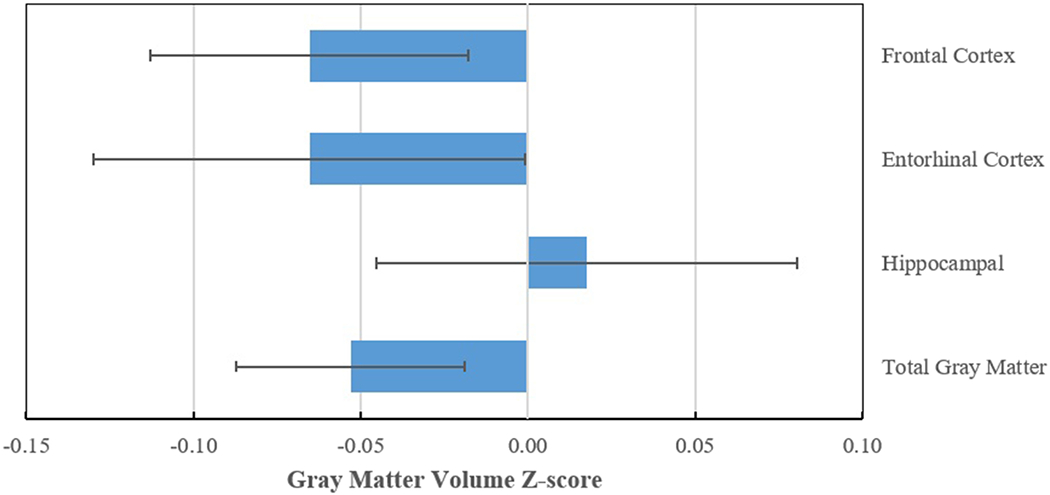
Television viewing and brain volume. Difference in brain MRI z-score per 1 standard deviation increase of television viewing. Figures reflect values adjusted for age, sex, race, education, intracranial volume, site, and physical activity.

For each standard deviation (SD) increase in mean television viewing (1 hour and 40 minutes), there was a small but significant difference in gray matter volume z-score for the regions of interest (Figure 2) except for the hippocampus. For total gray matter, the z-score was 0.053 less per SD increase in television viewing (*p* = 0.003). For both the frontal cortex and entorhinal cortex, the gray matter volume z-score was 0.065 less per SD increase in television viewing (*p* = 0.01 for frontal cortex; *p* = 0.05 for entorhinal cortex).

In sensitivity analyses, the observed association of television viewing with frontal cortex (β = −0.712; *p* = 0.03) and total gray matter (β = −1.836; *p* = 0.01) persisted after additional adjustment for smoking status, alcohol consumption, body mass index, blood pressure, and depression. There were no observed interactions of race, sex, or age with respect to television viewing and gray matter volume (all*p* > 0.05).

## Discussion

We sought to examine television viewing patterns over 20 years to determine associations between this sedentary behavior and gray matter volume in midlife. In this population-based sample of multiracial adults, greater long-term television viewing was negatively associated with gray matter volume in regions vulnerable to age-related atrophy. These findings persisted after controlling for the effects of demographics, lifestyle behaviors, and medical co-morbidities. Notably, television viewing remained associated with gray matter volume after accounting for physical activity, suggesting that this sedentary behavior may impart a unique risk with respect to brain health.

A growing body of research has detailed the benefits of physical activity for preserving gray matter volume (Dougherty et al. 2016; Erickson et al. 2014), mitigating cognitive decline (Yaffe et al. 2001), and reducing dementia risk (Barnes & Yaffe 2011; Hamer & Chida 2009). Studies have also shown the negative effect of sedentary behavior on cognitive health in older adulthood (Falck et al. 2017; Wheeler et al. 2017), however, it is less clear whether sedentary activities influence measures of brain health (e.g., brain volume) (Voss et al. 2014). Siddarth and colleagues reported preliminary relationships between high sedentary time in older adults and decreased gray matter volume within medial temporal lobe regions (e.g., entorhinal cortex) (Siddarth et al. 2018); however, no significant associations were reported in a similar sample of mid-late aged adults (Arnardottir et al. 2016). In a large study that leveraged data from the UK Biobank, physical activity, but not sedentary time, was related to gray matter volume in older adulthood (Raichlen et al. 2019). These prior studies often did not consider the type of sedentary activity (e.g. television viewing vs newspaper reading) but used a cumulative measure of time spent sitting. In the context of cognitive and brain health, not all sedentary behaviors are equal; non-stimulating sedentary activities such as television viewing are linked to greater risk of developing cognitive impairment (Fancourt & Steptoe 2019; Hoang et al. 2016; Lindstrom et al. 2005), whereas cognitively stimulating sedentary activities (e.g., reading, computer and board games) are associated with maintained cognition and reduced likelihood of dementia (Verghese et al. 2006; Verghese et al. 2003). In fact, a recent study found participation in cognitively stimulating sedentary activities (e.g., board games, puzzles, computer games) was associated with increased gray matter volume in midlife (Schultz et al. 2015). Therefore, the contradicting evidence to date may be due in part to lack of differentiation between non-stimulating sedentary activities and cognitively stimulating sedentary activities. Considering the contextual differences in varying sedentary behaviors appears to be critical when investigating cognitive and brain health.

Prolonged television viewing is one of the most common leisure-time sedentary activities among adults (Clark et al. 2009). In our sample, television viewing averaged 2.5 hours throughout early to mid-adulthood. This mirrors population data from the previous two decades which describe US adults spending 2-3 hours/day partaking in this sedentary activity (Yang et al. 2019). In our adjusted models, we report long-term television viewing was negatively associated with frontal cortex, entorhinal cortex, and total gray matter volume in midlife. Considering the effect estimates, a 1 hour greater mean television time was associated with approximately a 0.5% reduction in gray matter volume which is similar to the annual rate of atrophy throughout mid-late adulthood (Raz et al. 2005; Villemagne et al. 2013).

Because brain atrophy becomes apparent in midlife (Raz et al. 2005; Resnick et al. 2003; Villemagne et al. 2013), and this decline is associated with future cognitive impairment (Allison et al. 2019; Driscoll et al. 2009), our findings raise the question if reducing television viewing (or other non-stimulating sedentary behaviors) could preserve total gray matter volume and protect against future cognitive decline. Examining our regions of interest, the negative effect of television viewing on frontal cortex volume, but not hippocampal volume, extend upon our group’s previous work which reported prospective relationships between long-term patterns of television viewing and cognitive function (Hoang et al. 2016). In that large study from CARDIA, we found that participants who reported greater television viewing throughout early adulthood performed worse on midlife cognitive tests of executive function but not on memory (Hoang et al. 2016). Throughout the literature, there is general consensus that executive function primarily resides in frontal cortices (Fuster 2001), whereas memory formation relies largely on the hippocampus (Burgess et al. 2002). Our current findings build upon the previous CARDIA study and provide insight into a potential neurobiological mechanism through which high television viewing impacts midlife cognition.

We observed a weak correlation between television viewing and physical activity, replicating what has been observed in similar populations (Dunstan et al. 2012; Hoang et al. 2016; Katzmarzyk et al. 2009). Because sedentary behavior and physical activity are separate behavior phenotypes (Ainsworth et al. 2000), that have shown to impart unique risk of disease (Dunstan et al. 2012; Katzmarzyk et al. 2009), it is necessary to identify their independent contributions in order to understand the consequences of these lifestyle behaviors on gray matter volume. In our sample of middle-aged community dwelling adults, accounting for physical activity did not eliminate the negative association between television viewing and gray matter volume. Our findings, along with that of others (Ekelund et al. 2016; Hoang et al. 2016) suggest that television viewing, independent of physical activity, plays a role in brain, cognitive, and overall health.

There have been several proposed mechanisms for how sedentary behaviors may impact brain volume including endogenous growth factors, neuroendocrine regulation, and cerebral vascular health (Voss et al. 2014). Sedentary behaviors negatively influence several indices of neurovascular function such as central artery elasticity (Nualnim et al. 2011), and cerebral perfusion (i.e. blood flow) (Zlatar et al. 2019) which may precipitate brain volume decline. Decreasing sedentary behavior and increasing physical activity is known to enhance cardiovascular function (Seals et al. 1984) which promotes peripheral arterial health (Seals et al. 2008). These vascular adaptations may lead to improved cerebral blood flow regulation which in turn may mitigate brain atrophy (Barnes 2015).

The CARDIA Brain MRI ancillary study is a well-characterized diverse sample of community dwelling middle-aged adults with over 20 years of longitudinal data. However, there are study limitations to consider. Future studies that assess changes in gray matter volume are needed to better elucidate causality and directionality of the observed relationships. Although our models were adjusted for several important covariates, there are additional lifestyle habits that may accompany television viewing such as diet, sleep, and social factors that we were unable to control for. Further, while mobility impairments would be atypical for the age range of our participants, functional status was not measured and therefore may have influenced the results. Additionally, television viewing and physical activity were assessed through questionnaires, which might be subject to biases.

## Conclusion

There is a need to identify modifiable behaviors, such as excessive television viewing, that may be targeted prior to the development of cognitive impairment in an effort to promote healthy brain aging, particularly given current trends in television viewing and binge-watching behaviors (Flayelle et ah 2019). Our study provides evidence that within a multiracial middle-aged cohort, greater amounts of time spent viewing television throughout early to mid-adulthood was associated with reduced gray matter volume in midlife. This is an important finding as it is now well accepted that the neurobiology of dementia, including brain atrophy, begins during midlife, a period were modifiable behaviors, such as excessive television viewing, can be targeted and reduced in an effort to promote healthy brain aging.

## Figures and Tables

**Table 1:** Characteristics of study participants

	Overall television viewing (N = 599)			*p*-value
Baseline variables n (%) or mean (SD)	Low television N = 45 (7.5%)	Moderate television N = 473 (79.0%)	High television N = 81 (13.5%)	
Age (years)	51.0 (3.4)	50.2 (3.5)	49.6 (3.5)	0.08
Female	26 (57.8%)	246 (52.0%)	34 (42.0%)	0.16
Black	6 (13.3%)	192 (40.6%)	66 (81.5%)	< 0.001
Education (years)	15.9 (2.4)	15.0 (2.6)	13.1 (1.9)	< 0.001
BMI (kg/m2)	27.0 (5.9)	29.4 (5.7)	29.9 (5.5)	0.004
Current Smoker	1 (2.2%)	67 (14.2%)	33 (42.3%)	< 0.001
Alcohol Consumed (mL/day)	8.9 (13.0)	12.9 (20.3)	20.0 (48.4)	0.55
Systolic blood pressure (mm Hg)	113.3 (11.9)	117.5 (14.2)	125.2 (16.2)	< 0.001
Current Diabetes	2 (4.4%)	40 (8.5%)	10 (12.5%)	0.29
Current Depression	2 (4.4%)	57 (12.1%)	27 (33.3%)	< 0.001
Total physical activity (EU/week)	370.7 (218.4)	354.7 (270.0)	240.1 (224.5)	< 0.001

Low = television viewing ≤1 SD from the mean; Moderate = television viewing 1 SD < mean > 1 SD; High = television viewing ≥ 1 SD from the mean

**Table 2:** Linear regression estimates of association between 20-year average television viewing time and brain MRI gray matter among 599 adults.

Brain MRI	Unadjusted			Model 1			Model 2		
	B (SE)	β	*p*-value	B (SE)	β	*p*-value	B (SE)	β	*p*-value
Frontal Cortex	−2.37 (0.51)	−0.11	<0.001	−0.77 (0.30)	−0.04	0.01	−0.78 (0.30)	−0.04	0.01
Entorhinal Cortex	−71.18 (14.66)	−0.12	<0.001	−23.83 (12.01)	−0.04	0.05	−23.84 (12.01)	−0.04	0.05
Hippocampal	−60.80 (20.34)	−0.07	0.003	11.44 (16.02)	0.01	0.48	8.76 (16.04)	0.01	0.59
Total Gray Matter	−8.31 (1.58)	−0.13	<0.001	−2.09 (0.69)	−0.03	0.003	−2.09(0.69)	−0.03	0.003

*MRI* Magnetic resonance imaging, *B*, unstandardized beta coefficient; *SE*standard error, *β*, standardized beta coefficient

Model 1: adjusted for age, sex, race, education, total intracranial volume, and site

Model 2: Model 1 + adjusted for total physical activity
